# Transcranial Direct Current Stimulation of the Dorsolateral Prefrontal Cortex for Treatment of Neuropsychiatric Disorders

**DOI:** 10.3389/fnbeh.2022.893955

**Published:** 2022-05-25

**Authors:** Qing Li, Yu Fu, Chang Liu, Zhiqiang Meng

**Affiliations:** ^1^Medical School, Kunming University of Science and Technology, Kunming, China; ^2^Shenzhen Key Laboratory of Drug Addiction, Brain Cognition and Brain Disease Institute, Shenzhen Institute of Advanced Technology, Chinese Academy of Sciences, Shenzhen, China; ^3^Shenzhen Key Laboratory of Viral Vectors for Biomedicine, Brain Cognition and Brain Disease Institute, Shenzhen Institute of Advanced Technology, Chinese Academy of Sciences, Shenzhen, China; ^4^Shenzhen-Hong Kong Institute of Brain Science, Shenzhen Fundamental Research Institutions, Shenzhen, China; ^5^CAS Key Laboratory of Brain Connectome and Manipulation, Shenzhen Institute of Advanced Technology, Chinese Academy of Sciences, Shenzhen, China

**Keywords:** non-invasive neuromodulation, dorsolateral prefrontal cortex (DLPFC), schizophrenia, addiction, depression, psychiatric disease

## Abstract

**Background:**

The dorsolateral prefrontal cortex (DLPFC) is a key node of the frontal cognitive circuit. It is involved in executive control and many cognitive processes. Abnormal activities of DLPFC are likely associated with many psychiatric diseases. Modulation of DLPFC may have potential beneficial effects in many neural and psychiatric diseases. One of the widely used non-invasive neuromodulation technique is called transcranial direct current stimulation (or tDCS), which is a portable and affordable brain stimulation approach that uses direct electrical currents to modulate brain functions.

**Objective:**

This review aims to discuss the results from the past two decades which have shown that tDCS can relieve clinical symptoms in various neurological and psychiatric diseases.

**Methods:**

Here, we performed searches on PubMed to collect clinical and preclinical studies that using tDCS as neuromodulation technique, DLPFC as the stimulation target in treating neuropsychiatric disorders. We summarized the stimulation sites, stimulation parameters, and the overall effects in these studies.

**Results:**

Overall, tDCS stimulation of DLPFC could alleviate the clinical symptoms of schizophrenia, depression, drug addiction, attention deficit hyperactivity disorder and other mental disorders.

**Conclusion:**

The stimulation parameters used in these studies were different from each other. The lasting effect of stimulation was also not consistent. Nevertheless, DLPFC is a promising target for non-invasive stimulation in many psychiatric disorders. TDCS is a safe and affordable neuromodulation approach that has potential clinical uses. Larger clinical studies will be needed to determine the optimal stimulation parameters in each condition.

## Introduction

Neuropsychiatric disorders are combinations of psychiatric and neurologic malfunction that deal with mental disorders, including degenerative diseases, addictions, mood disorders, neurotic disorders, etc. Current treatments of neuropsychiatric diseases mainly include drug therapy, physical therapy and psychotherapy. Common physical therapies included electroconvulsive treatment (ECT), deep brain stimulation (DBS), transcranial magnetic stimulation (TMS), transcranial direct current stimulation (tDCS), etc. Among these techniques, tDCS becomes an increasingly employed clinically due to its economical, convenient, non-invasive and mild side effects. However, current dilemma in using tDCS as a option of clinical treatment is that there is no common standard, and the therapeutic effects vary from case to case.

In this review, we discussed: (1) the mechanism of tDCS and the application of tDCS technique in clinical research, focusing on five types of psychiatric disorders; (2) and the potential therapeutic brain target DLPFC.

## An Overview of Transcranial Direct Current Stimulation Technique

Accumulating knowledge has supported that transcranial direct current stimulation (tDCS) can relieve symptoms of various diseases, including pain (Wrigley et al., 2013), depression (Sharafi et al., 2019), schizophrenia (Brunelin et al., 2012a), attention deficit disorder (Cosmo et al., 2015), drug addiction (da Silva et al., 2013), and anxiety disorder (Heeren et al., 2017). In recent years, tDCS has been widely used in clinical research due to the advantages mentioned above. tDCS is a non-invasive brain stimulation technique that uses low-intensity direct current (1–2 mA) to modulate cortical activity (Woods et al., 2016). A common tDCS stimulator consists of a controller to generate a constant current, and at least one pair of stimulation electrodes to attach to the surface of the scalp. Although there is no uniform standard for stimulation parameters in clinical studies, electrodes of 20–35 cm^2^, with application of 1–2 mA currents, 20- or 30-min stimulation duration for one session with one or multiple sessions through a certain period have been employed in a large body of studies.

The activity of the brain is based on the electrical activity of neurons. It is believed that tDCS may modulate the brain activity at different scales. First, from a macro perspective, tDCS likely modulate the brain activity via changing the cortical excitability directly. In general, anodal stimulation depolarizes neurons, whereas cathodal stimulation hyperpolarizes neurons (Purpura and McMurtry, 1965; Bikson et al., 2004). In addition, tDCS may regulate the activity of neural networks by influencing other brain regions associated with the target brain region. It has been suggested that neuronal networks were more sensitive than single neuron in the weak electric field (Francis et al., 2003). By using resting-state functional magnetic resonance imaging (fMRI) technique, it has been found that anode tDCS intensified the functional connection among the thalamus, the temporal lobe and the left caudate nucleus (Dalong et al., 2020). At the neuronal levels, tDCS has been shown to modulate the neural oscillations. McDermott et al. (2019) reported that anode tDCS increased spontaneous activity in the theta (4–7 Hz) and alpha (9–14 Hz) bands in prefrontal and occipital cortices in a flanker task. Finally, from the molecular perspective, tDCS may modulate neurotransmitter release to regulate synaptic plasticity. For example, long-term potentiation (LTP) which was observed after anodal tDCS coupling with synaptic activation (Fritsch et al., 2010). Another study found that the effects of tDCS may be related to the polarity-specific changes in neurotransmitter concentrations. Anodal tDCS caused locally reduced GABA concentrations while cathodal stimulation caused reduced glutamatergic neuronal activity with a highly correlated increase in GABA concentration (Stagg et al., 2009). Liebetanz et al. (2002) showed that, dextromethorphan, an antagonist of N-Methyl-D-Aspartic Acid receptors (NMDAR, receptors that are involved in synaptic plasticity regulation), suppressed the post-stimulation effects of both anode and cathode stimulation.

In order to recommend this convenient technique as a powerful therapeutic strategy, a remarkable effort is still needed to further understand how tDCS modulate the brain activity.

## Dorsolateral Prefrontal Cortex Is a Target for Non-Invasive Stimulation in Neuropsychiatric Diseases

**FIGURE 1:**
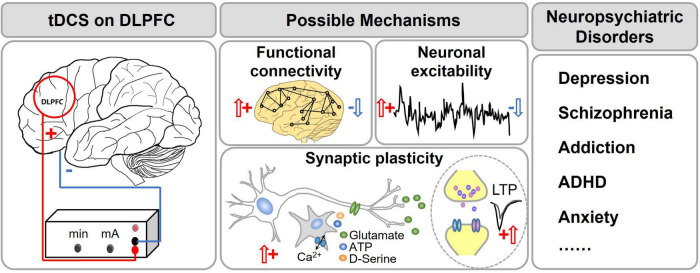
tDCS of the dorsal lateral prefrontal cortex (DLPFC) for treatment of neuropsychiatric disorders. The red circle shows the DLPFC. It is the center for higher brain functions such as working memory, executive function, attention, etc. Dysfunction of DLPFC was found in many psychiatric disorders such as schizophrenia, depression, ADHD, etc. tDCS of DLPFC has become a popular treatment option for these disorders. It has been proposed that tDCS changes the functional connectivity, neuronal excitability and synaptic plasticity of the related brain regions.

One of the most common cortical targets for tDCS is the dorsolateral prefrontal cortex (DLPFC; Figure 1). DLPFC is a structurally and functionally heterogeneous region (Glasser et al., 2016), and is closely related with cognitive functions [attention (Vossel et al., 2014; Bidet-Caulet et al., 2015), decision-making (Philiastides et al., 2011; Rahnev et al., 2016), working memory (Barbey et al., 2013), and emotion regulation (Shahani and Russell, 1969; Buhle et al., 2014; Frank et al., 2014)]. The DLPFC is located in the middle frontal gyrus, and it is a part of the prefrontal cortex (PFC) which regulates the marginal reward area, and involves in higher executive function and impulsive behaviors (Fitzpatrick et al., 2013; Xu et al., 2017). The left DLPFC connects to the primary motor area, primary sensory area, etc. It mainly participates in pain perception and emotional cognitive processing through a top-down neural network (Koenigs and Grafman, 2009; Vaseghi et al., 2015). The right DLPFC is selectively involved in processing pessimistic, negative emotions and mediates vigilance and arousal (Hecht, 2010). DLPFC has become an important target in the treatment for mental disorders.

A large number of studies have shown that tDCS targeting at DLPFC can alleviate a variety of neuronal and psychiatric diseases symptoms. For example, anode tDCS (2 mA) can reduce the pain caused by multiple sclerosis (Ayache et al., 2016). Anode stimulation of the right DLPFC, and cathode at the left DLPFC improved the risk preference of the subjects (Yang et al., 2017). Studies have shown that anodal tDCS stimulation of left DLPFC could decrease negative emotions and improve cognitive control (Pena-Gomez et al., 2011). Here, we summarize and discuss perspectives of the parameters and effects of tDCS targeting DLPFC in the treatment of different types of neuropsychiatric disorders.

### Depression

Depression (also known as depressive disorder) is a mental disease that causes a persistent feeling of sadness and loss of interests, with high recurrence rate, disability rate and suicide rate. In general, it can be classified into major depression, bipolar disorder or treatment-resistant depression. Bipolar disorder, causing extreme mood swings that include emotional highs (mania or hypomania) and lows (depression). Treatment-resistant depression refers to no response to at least two different antidepressant treatments.

Twenty studies collected from PubMed were shown in Table 1. Majority of these studies have shown that tDCS targeting at DLPFC (mostly the left DLPFC) can significantly improve depression symptoms for a month or longer. All studies placed the anode electrodes on the left DLPFC and the cathode electrodes on the opposite side (right DLPFC or orbitofrontal region). 17 out of 20 studies reported improvement of depressive symptoms. Besides, tDCS also improved working memory and attention (Loo et al., 2012). Importantly, tDCS in combination with other treatments, such as an antidepressant drug (Brunoni et al., 2013b) or with computerized cognitive behavioral therapy (Welch et al., 2019), can reduce depressive symptoms even better than tDCS alone (Brunoni et al., 2013a). It is important to note that tDCS on DLPFC may have some side effects, such as mania, although this is not common (Loo et al., 2012). For the stimulation parameters, most studies have used a current of 2 mA, electrode sizes of 25–35 cm^2^, and a total of more than five sessions (see details in Table 1). Though various parameters have shown different effects on depression symptoms, most stimulation protocols with longer stimulation duration for one session and repeated sessions were shown to have therapeutic effects.

**TABLE 1:** Effects of DLPFC tDCS on depression.

References	Electrode montage		Electrode size (cm^2^)	Current intensity (mA)	Stimulation duration (min)	Stimulation sessions	Total sessions	Key findings
		
	Anode (+)	Cathode (−)						
Brunoni et al., 2017	DLPFC (F3)	DLPFC (F4)	/	2	30	1/day, 3 weeks + 1/week × 7 weeks	22	Have a significant effect, but it was inferior to escitalopram
Aparicio et al., 2019	DLPFC (F3)	DLPFC (F4)	25	2	30	1/day, 3 weeks, +1/week, 7 weeks	22	Reduced recurrence rate significantly
Moreno et al., 2020	DLPFC (F3)	DLPFC (F4)	/	2	30	1/day, 3 weeks, +1/week, 7 weeks	22	Reduced practice effects in processing speed, but no change in cognitive deficits
Palm et al., 2012	DLPFC (F3)	Orbitofrontal region	35	1/2	20	1/day, 4 weeks	20	No significant effect
Martin et al., 2013	DLPFC (F3)	Arm/opposite Side of track (F8) (two forms of tDCS)	35	2	20	1/week × 3 months + 1/2 weeks × 3 months	18	Reduced the recurrence rate for relapse significantly
Sampaio-Junior et al., 2018	DLPFC (F3)	DLPFC (F4)	25	2	30	1/day, 2 weeks + 2/other week, 6 weeks	16	Have a significant improvement
Brunoni et al., 2013b	DLPFC (F3)	DLPFC (F4)	25	2	30	1/day × 2 weeks + 1/2 weeks × 2	12	Improved mood significantly [tDCS + sertraline (50 mg/d)]
Welch et al., 2019	DLPFC (F3)	DLPFC (F4)	25	2	30	3/week × 4 weeks	12	Reduced depressive symptoms significantly (tDCS + computerized cognitive behavioral therapy)
Brunoni et al., 2014	DLPFC (F3)	DLPFC (F4)	25	2	30	1/day, 2 weeks	10	Reduced depressive symptoms significantly
Blumberger et al., 2012	DLPFC (F3)	DLPFC (F4)	35	2	20	1/day, 3 weeks	15	No significant effect
Loo et al., 2012	DLPFC (F3)	Orbitofrontal region (F8)	35	2	20	1/day, 3 weeks	15	Improved mood significantly
Loo et al., 2010	DLPFC (F3)	Orbitofrontal region	35	1	20	5 active + 5 active sessions	10	Improved overall depression significantly over 10 tDCS treatments, no between-group difference in the five-session, sham-controlled phase
					20	5 sham session + 5 active sessions	10	
Dell’Osso et al., 2012	DLPFC (F3)	Contralateral cortex	32	2	20	2/day × 5 days	10	Have a significant improvement
Sharafi et al., 2019	DLPFC (F3)	DLPFC (F4)	20	2	20	1/day, 2 weeks	10	Have a significant effect (lasted for 1 month after treatment)
Lin et al., 2021	DLPFC (F3)	DLPFC (F4)	35	2	20	2/day × 5 days	10	Improved unipolar and bipolar depression rapidly
Brunoni et al., 2011	DLPFC (F3)	DLPFC (F4)	35	2	20	2/day × 5 days	10	Improved depression for 1 week in MDD group and 1 month in BDD group
Rigonatti et al., 2008	DLPFC (F3)	Contralateral Superior orbital region	35	2	20	1/day × 10 days	10	Have a significant effect (similar to fluoxetine 20 mg/day for 6 weeks)
Boggio et al., 2008a	DLPFC (F3)	Contralateral Supraorbital area	35	2	20	1/day, 2 weeks	10	Reduced depression scores significantly (lasted for 1 month after treatment) after DLPFC tDCS compared to occipital and sham tDCS
Bennabi et al., 2015	DLPFC (F3)	Contralateral superior Orbital region	35	2	20	2/day × 5 days	10	No significant effect
Kumar et al., 2020	left DLPFC (F3) + right DLPFC (F4)	Iz	25	1	30	1/day, 2 weeks	10	No significant effect

*MDD, major depressive disorder; BDD, bipolar depressive disorder.*

### Schizophrenia

Schizophrenia is a chronic mental disorder. The most typical symptoms of schizophrenia include hallucinations and delusions, which are often referred to as positive symptoms. Schizophrenia may also experience negative symptoms, such as social withdrawal, anhedonia, hyperboulia, affective blunting and alogia (Carpenter et al., 2016). In recent years, clinical studies have shown that tDCS may be effective in reducing auditory hallucination symptoms in patients with schizophrenia. For example, a study reported that anode tDCS showed a significant increase in short- interval intracortical inhibition in the left motor cortex, but no change in intra-cortical facilitation (ICF) compared to sham stimulation (Gordon et al., 2019). Yoon et al. (2019) found that decreased functional network connectivity was negatively correlated with the increase of hallucinogenic behavior at baseline and was significantly enhanced after anode 2 mA tDCS. This may suggest that fronto-temporal tDCS may regulate abnormal hallucination-related functional network connectivity in patients with schizophrenia. Decline in insight is also one of the main symptoms of schizophrenia. Patients with insight deficits often fail to recognize that they are ill and may refuse treatment. Bose et al. (2014) found that 2 mA anode tDCS stimulation over left DLPFC and cathode over the left temporo-parietal junction, could improve the insight and decrease auditory hallucination symptoms in patients. However, no such effect was observed after 1 mA stimulation, which indicates that the current intensity of tDCS is a key factor (Hill et al., 2016). A combination of medication, physical therapy, and psychotherapy usually have a synergic effect. Non-invasive brain stimulation combined with physical therapy has been shown to improve motor performance and language function in stroke patients (Barros Galvao et al., 2014; Rubi-Fessen et al., 2015). Orlov et al. (2017) found that anode tDCS stimulation combined with cognitive behavioral training showed significant improvement in working memory and learning. However, Shiozawa et al. (2016) found that tDCS combined with cognitive training failed to produce a synergic effect in schizophrenia patients. This may due to the small sample size and the use of antipsychotics in patients (Orlov et al., 2017).

We summarized 28 studies using tDCS as a treatment strategy for schizophrenia in Table 2. Overall, tDCS improved both positive syndromes and negative syndromes in patients with schizophrenia. Only two studies showed no significant improvement after tDCS. For the electrodes positions, in 26 out of 28 studies placed the anode in the left DLPFC (F3) or a point midway between F3 and FP1 and the cathode in the right hemisphere (left temporoparietal junction, FP2, or right contralateral superior orbital region). 20 out of 28 studies used 25–35 cm^2^ electrodes. For stimulating current intensity, 26 studies used 2 mA current, only 1 study used 1 mA current, and 1 study used both 1 mA and 2 mA current. For stimulation duration, 26 studies used 20 min/session, 1 study used 30 min/session, and 1 study used 15 min/session. All studies adopted multiple stimulation sessions (from 5 to 20 sessions), only two studies used one single session of tDCS. Most multiple sessions of tDCS brought a better curative effect, pointing to a repeated application of tDCS as therapeutic strategy. In studies with one single session of tDCS, 2 mA but not 1 mA was shown to induce a positive effect. Taken together, 2 mA multi-session anodal tDCS of the left DLPFC or left temporoparietal junction area has the most potential to improve symptoms in patients with schizophrenia.

**TABLE 2:** Effects of tDCS of DLPFC on schizophrenia.

References	Electrode montage		Electrode size (cm^2^)	Current intensity (mA)	Stimulation duration (min)	Stimulation sessions	Total sessions	Key findings
		
	Anode (+)	Cathode (−)						
Weickert et al., 2019	Right DLPFC (F4)	Left Temporoparietal junction	35	2	20	1/day, 4 weeks	20	Improved language-based working memory after 2 weeks, and oral fluency after 2 and 4 weeks significantly
Bose et al., 2015	Right DLPFC (a point midway between F4 and FP2)	Right left temporoparietal junction	35	2	20	2/day × 9 days	18	Right DLPFC tDCS reduced auditory hallucinations, but no change after left DLPFC tDCS
	Left DLPFC (a point midway between F3 and FP1)	Left temporoparietal junction						
Fitzgerald et al., 2014	Left DLPFC (F3)	Left temporo-parietal junction (unilaterally F3/TP3 or bilaterally F3 + F4/TP3 + TP4)	35	2	20	1/day, 3 weeks	15	No significant effect
Brunelin et al., 2012a	Left DLPFC (F3)	Left temporo-parietal cortex	35	2	20	2/day × 5 days	10	Reduced AVH significantly (lasted for 3 months after treatment), improved negative symptoms
Brunelin et al., 2012b	Left DLPFC (F3)	Left temporo-parietal cortex	35	2	20	2/day × 5 days	10	Have a significant effect (lasted for 3 months after treatment)
Shiozawa et al., 2013	Left DLPFC (F3)	Cathode: right DLPFC (F4)	35	2	20	1/day × 10 days	10	Improved catatonic symptoms significantly (remained for 4 weeks after treatment)
Jacks et al., 2014	Left DLPFC (F3)	Left temporo-parietal cortex	/	2	20	2/day × 5 days	10	Improved mood, feelings of hope, and fewer AVH, but no change in PANSS score
Jeon et al., 2018	Left DLPFC (F3)	Right DLPFC (F4)	25	2	30	1/day, 2 weeks	10	Improved working memory over time
Valiengo et al., 2020	Left DLPFC (F3)	Left temporoparietal junction	35	2	20	2/day × 5 days	10	Improved PANSS score significantly
Narayanaswamy et al., 2014	Left DLPFC (F3)	Cathode: left temporo-parietal cortex	/	2	20	2/day × 5 days	10	Improved in negative symptoms and AVH significantly (lasted for 6 months after treatment)
Palm et al., 2016	Left DLPFC (F3)	Right contralateral superior orbital region	35	2	20	1/day, 2 weeks	10	Improved negative and positive symptoms significantly
Palm et al., 2013	Left DLPFC	Right contralateral superior orbital region	/	2	20	1/day × 10 days	10	Improved negative and positive symptoms significantly
Brunelin et al., 2015	Left DLPFC	Left temporoparietal junction	35	2	20	2/day × 5 days	10	Reduced AVH significantly
Bose et al., 2014	Left DLPFC (a point midway between F3 and FP1)	Left temporoparietal junction	35	2	20	2/day × 5 days	10	Improved insight and reduced AVH
Mondino et al., 2015	Left DLPFC (a point midway between F3 and FP1)	Left temporoparietal junction	35	2	20	2/day × 5 days	10	Reduced AVH significantly
Mondino et al., 2016	Left DLPFC (a point midway between F3 and FP1)	Left temporoparietal junction	35	2	20	2/day × 5 days	10	Improved in negative symptoms and AVH significantly
Nawani et al., 2014a	Left DLPFC (a point midway between F3 and FP1)	Left temporoparietal junction	/	2	20	2/day × 5 days	10	Have a significant reduction in AHRS score
Rakesh et al., 2013	Left DLPFC (a point midway between F3 and FP1)	Left temporoparietal junction	/	2	20	2/day × 5 days	10	Reduced AVH significantly
Shenoy et al., 2015	Left DLPFC (a point midway between F3 and FP1)	Left temporoparietal junction	/	2	20	2/day × 5 days	10	Reduced AVH significantly (lasted for 1 month after treatment)
Chang et al., 2019	Left DLPFC (a point midway between F3 and FP1)	Left temporo-parietal junction	35	2	20	2/day × 5 days	10	Improved overall symptoms
Chang et al., 2020	Left DLPFC (a point midway between F3 and FP1) + right DLPFC (a point midway between F4 and Fp2)	Forearms	35	2	20	2/day × 5 days	10	Reduced AVH significantly (lasted for 3 months after treatment)
Homan et al., 2011	Left temporo-parietal cortex	Right supraorbital area	35	1	15	1/day, 2 weeks	10	Reduced AVH significantly (lasted for 6 weeks after treatment)
Praharaj et al., 2015	Left DLPFC (F3)	Midway between T3 and P3	25	2	20	1/day × 5 days	5	Reduced AVH temporarily
Nawani et al., 2014b	Left prefrontal	Left temporoparietal	/	2	20	1/day × 5 days	5	Reduced AVH significantly
Smith et al., 2015	Left DLPFC (F3)	Right contralateral superior orbital region	5.08	2	20	1/day × 5 days	5	Improved memory, attention, and cognitive function significantly
Frohlich et al., 2016	Left DLPFC (a point midway between F3 and FP1)	Left temporoparietal junction	35	2	20	1/day × 5 days	5	Reduced AVH, but overall symptoms did not change significantly
Schilling et al., 2021	Left DLPFC (F3)	FP2	25	2	20	1/day	1	No enhancement in executive functions
Hoy et al., 2014	Left DLPFC (F3)	Right contralateral superior orbital region	35	1/2	20	1/day	1	Improved cognitive performance only after 2 mA tDCS

### Addiction

Addiction is a chronic brain disease characterized by compulsive use of drugs, with loss of self-control and a high relapse rate (Berke and Hyman, 2000; Preller et al., 2013). Patients may experience negative emotions during withdrawal, such as sadness, restlessness, subdued pleasure. The relapse tendency indicates that a solid memory of drugs, a pathological memory, also called drug memory formed in addiction patients (Boning, 2009; Nestler, 2013). Drug memory is signaled by dynamic neuronal activity patterns in the brain areas such as prefrontal cortex, hippocampus and the ventral tegmental area (VTA; Berke and Hyman, 2000). Drugs increase the activity of VTA dopaminergic neurons as well as the concentration of dopamine in the projection area (Hyman and Malenka, 2001; Pierce and Kumaresan, 2006). The downstream targets of VTA dopaminergic neurons mainly includes ventral striatum, which is responsible for processing reward information, and prefrontal cortex, which is responsible for higher brain functions such as decision making, executive function, etc. (Robbins and Everitt, 2002; Hyman et al., 2006). Reward related perception and executive function can be modulated by the release of dopamine in the frontal lobe (Goldstein and Volkow, 2002).

Many studies have shown that tDCS can significantly relieve the symptoms of addictions (such as craving for cocaine, cigarette, alcohol, etc.). Bilateral DLPFC tDCS stimulation reduced cocaine craving with a linear decrease within 4 weeks, and improved anxiety symptoms and overall quality of life in patients (Batista et al., 2015). In addition to cocaine, tDCS stimulation can also reduce cravings for alcohol and cigarettes. Klauss et al. (2018b) showed that bilateral DLPFC tDCS stimulation significantly reduced alcohol cravings and reduced recurrence rates. Fecteau et al. (2014) found that the number of cigarettes consumed decreased significantly after bilateral DLPFC stimulation, and the effect could last for 4 days after the stimulation. Besides, non-substance addiction, such as food addiction, gambling addiction and internet addiction, shows executive function (such as decision-making and risk- taking processes) and working memory deficits similar to those in drug addiction (Fernandez-Serrano et al., 2010; Marazziti et al., 2014; Potenza, 2014). Studies have shown that anode tDCS stimulation of the right DLPFC decreased craving and negative emotions in addicted internet gaming players (Wu et al., 2020). Fregni et al. (2008b) found that the bilateral tDCS stimulation, left anode/right cathode or right anode/left cathode, reduced the food craving as well.

In Table 3, we summarized 21 studies evaluated tDCS treatment in substance addiction. Four studies didn’t observe any improvement after tDCS treatment. All other studies showed tDCS reduced craving, improved behavioral control and reduced likelihood of relapse. Most studies used 25–35 cm^2^ electrodes. For stimulating current intensity, 14 studies used 2 mA current, and 7 studies used a lower current. For stimulation duration, 4 studies used 10∼15 min/session, other studies used 20 min/session. There are 18 studies applied stimulation sessions from 1 to 4, and three of these studies showed no positive effects the rest studies used stimulation sessions from 5 to 20, which induced significant improvement of addiction symptoms except for one study. Roughly half of the studies placed anodal electrode on the right DLPFC, and the other half on the left. A couple of studies tried both montages. Together, tDCS of the DLPFC (left and/or right) has the potential to improve symptoms and reduce craving in substance addiction.

**TABLE 3:** Effects of DLPFC tDCS on addiction behaviors.

References	Substance	Electrode montage		Electrode size (cm^2^)	Current intensity (mA)	Stimulation duration (min)	Stimulation sessions	Total sessions	Key findings
		
		Anode (+)	Cathode (−)						
Ghorbani Behnam et al., 2019	Smoking	Left DLPFC (F3)	Right DLPFC (F4)	35/100	2	20	1/day, 4 weeks	20	Reduced smoking addiction only in active group (20 sessions, 12 weeks). The effect was similar to 300 g bupropion
							1/day, 2 weeks + 1/week, 10 weeks	20	
Mondino et al., 2018	Smoking	Right DLPFC (F4)	Left occipital region	35/100	2	20	2/day × 5 days	10	Reduced smoking cue related craving significantly and increased brain reactivity in the right posterior cingulate cortex
Klauss et al., 2018a	Cocaine	Right DLPFC (F4)	Left DLPFC (F3)	35	2	20	1/every other day	10	No significant effect
Klauss et al., 2018b	Alcohol	Right DLPFC (F4)	Left DLPFC (F3)	35	2	20	1/every other day	10	Reduced alcohol cravings and recurrence rates significantly
da Silva et al., 2013	Alcohol	Left DLPFC (F3)	Contralateral (right) supradeltoid area	35	2	20	1/day × 5 days	5	Improved depressive symptoms and reduced alcohol craving
Holla et al., 2020	Alcohol	Right DLPFC (F4)	Left DLPFC (F3)	35	2	20	1/day × 5 days	5	Increase the global efficiency of brain networks significantly with a concurrent significant reduction in global clustering
Batista et al., 2015	Cocaine	Left DLPFC (F3)	Right DLPFC (F4)	35	2	20	1/every other day	5	Decreased craving for crack-cocaine use, anxiety, and improved quality of life
Vitor de Souza Brangioni et al., 2018	Smoking	Left DLPFC (F3)	Right supra-orbital area	35	1	20	1/day × 5 days	5	Reduced cigarette consumption up to 4-weeks post-intervention coupled with high motivation to quite
Boggio et al., 2009	Smoking	Left DLPFC (F3)	Right DLPFC (F4)	35/100	2	20	1/day × 5 days	5	A significant cumulative effect on modifying smoking cue-provoked craving, with significant decrease in the number of cigarettes
Fecteau et al., 2014	Smoking	Right DLPFC (F4)	Left DLPFC (F3)	35	2	30	1/day × 4 days	4	Decreased the amount of smoking significantly (lasted for 4 days after stimulation)
den Uyl et al., 2017	Alcohol	Left DLPFC (F3)	Right DLPFC (F4)	35/100	2	20	1/day × 4 days	4	No significant effect
den Uyl et al., 2016	Alcohol	Contralateral supraorbital region	Left DLPFC (F3)	35	1	15	1/day × 3 days	3	Decreased cue-induced craving (but not overall craving) on post assessment, but no effects on cognitive bias modification (CBM)
Alghamdi et al., 2019	Smoking	Left DLPFC (F3)	Right DLPFC (F4)	25	1.5	20	1/day × 3 days	3	No significant effect
Boggio et al., 2008b	Alcohol	Right DLPFC (F4)	Left DLPFC (F3)	35	2	20	1/day	1	Reduced alcohol craving significantly in two active stimulation groups, and alcohol craving did not increase further after treatment
		left DLPFC (F3)	Right DLPFC (F4)						
den Uyl et al., 2015	Alcohol	Left DLPFC (F3)	Contralateral supraorbital region	35	1	10	1/day	1	Anodal tDCS over the DLPFC reduced alcohol craving significantly, stimulation of the IFG did not decrease craving
		Right inferior frontal gyrus (IFG)	Contralateral supraorbital region						
Wietschorke et al., 2016	Alcohol	Right DLPFC (F4)	Left DLPFC (F3)	35	1	20	1/day	1	Reduced alcohol craving
		Left DLPFC (F3)	Right DLPFC (F4)						
Fregni et al., 2008a	Smoking	Left DLPFC (F3)	Contralateral hemisphere	35/100	2	20	1/day	1	Both anodal and cathodal tDCS to left DLPFC significantly reduced craving
		Right DLPFC (F4)	Contralateral hemisphere						
Xu et al., 2013	Smoking	Left DLPFC (F3)	Right supra-orbital area	35	2	20	1/day	1	Reduced negative emotions, but no reduction in cigarette craving
Kroczek et al., 2016	Smoking	Left DLPFC (F3)	Contralateral right supradeltoid area	35	2	15	1/day	1	No significant effect
Falcone et al., 2016	Smoking	Left DLPFC (F3)	Right supra-orbital area	25	1	20	1/day	1	Increased latency to smoke and decreased the total number of cigarettes smoked significantly
Gorini et al., 2014	Cocaine	Left DLPFC (F3)	Right DLPFC (F4)	32	1.5	20	1/day	1	Increased safe behavior after right DLPFC anodal stimulation, increased risk-taking behavior after left DLPFC anodal stimulation
		Right DLPFC (F4)	Left DLPFC (F3)						

### Attention Deficit Hyperactivity Disorder

Attention Deficit Hyperactivity Disorder (ADHD) is a brain disorder that characterized with inattention, impulsivity, hyperactivity and learning disabilities. ADHD mainly occurs in primary and middle schools (6–17 years old), and the prevalence is as high as over 6% (Rowland et al., 2015). The prevalence of ADHD is higher in boys than girls, and the risk for premature infants is also higher (Polanczyk et al., 2015). Neuroimaging studies have shown that the symptoms in ADHD patients may be related to abnormalities in fronto–striato–cerebellar neural circuit, especially the prefrontal lobe (Cubillo et al., 2012; Christakou et al., 2013). Specifically, the activity of bilateral striato-thalamus, left DLPFC and superior parietal cortex was significantly reduced in ADHD patients, and the activity of precuneus was significantly increased (Hart et al., 2013). Adults with childhood ADHD showed reduced activation in bilateral inferior prefrontal cortex, caudate and thalamus compared to controls. Neuro-functional abnormalities in ADHD patients are likely to persist from childhood to adulthood (Cubillo et al., 2010). fMRI studies also showed that striatum activation was abnormal in ADHD children (Durston et al., 2003).

In recent years, tDCS has been considered to have an ameliorative effect on ADHD symptoms. Studies have shown that 1 mA anode tDCS of the left DLPFC improved the executive function in adolescent ADHD patients. After tDCS, they showed better inhibitory control, interference control, working memory and cognitive flexibility (Nejati et al., 2020). Blair’s research showed that inhibitory control is the main executive problem for adolescents with ADHD, and the problems with inhibitory control will lead to dysfunctions in memory, emotion regulation and other executive functions (Blair and Razza, 2007). tDCS improves the symptoms not only in adolescent patients, but also in adult ADHD patients. Left DLPFC tDCS in adult ADHD patients improved the impulsiveness symptoms (Allenby et al., 2018), and bilateral tDCS (anode over right DLPFC, cathode over left DLPFC) improved the inattention symptoms (Cachoeira et al., 2017). Only several studies were collected here which were shown in Table 4. All these studies targeted left DLPFC with anodal stimulation. One out of six studies (used a single session protocol) showed negative results, and all the rest found tDCS improved ADHD related symptoms. The stimulation current was 1 mA or 2 mA, 1 session to 5 sessions in total. While the potential of tDCS of the DLPFC to treat ADHD is promising, the published studies are relatively fewer compared to other diseases.

**TABLE 4:** Effects of DLPFC tDCS on ADHD.

References	Electrode montage		Electrode size (cm^2^)	Current intensity (mA)	Stimulation duration (min)	Stimulation sessions	Total sessions	Key findings
		
	Anode (+)	Cathode (−)						
Soff et al., 2017	DLPFC (F3)	Vertex	3.14/12.5	1	20	1/day × 5 days	5	Improved inattention and impulsivity, and the effect lasted for 7 days
Cachoeira et al., 2017	DLPFC (F3)	DLPFC (F4)	35	2	20	1/day × 5 days	5	Improved inattention
Allenby et al., 2018	DLPFC (F3)	Supra-orbital area	25	2	20	3/week	3	Improved impulsivity symptoms acutely (conners continuous performance task) but not the stop signal task
Dubreuil-Vall et al., 2021	DLPFC (F3)	Contralateral Supraorbital region (Fp1 or Fp2)	3.14	2	30	1/day	1	Modulated reaction time and P300 amplitude in the Eriksen flanker task, but not in the stop signal task
	DLPFC (F4)							
Cosmo et al., 2015	DLPFC (F3)	Right DLPFC (F4)	35	1	20	1/day	1	No significant differences in behavioral performance
Gogler et al., 2017	DLPFC (F3)	Right contralateral Superior orbital region	25	2	20	1/day	1	Improved inattention

### Anxiety

Anxiety disorders are the most common form of emotional disorder characterized by nervousness, worry and fear. There are several types of anxiety disorders, including generalized anxiety disorder (GAD), Social anxiety disorder (SAD), post-traumatic stress disorder (PTSD), panic disorder (PD), obsessive compulsive disorder (OCD), agoraphobe and specific phobia. Studies have shown that OCD symptoms are related to the cortico-striato-thalamocortical circuitry, including DLPFC, orbital frontal lobe (OFC), medial prefrontal lobe (MPF), and anterior cingulate cortex (ACC; Del Casale et al., 2011; Fineberg et al., 2011). Striatal dysfunction may lead to hypothalamic gating problems and hyperactivity in the orbitofrontal cortex and anterior cingulate cortex in OCD patients (Milad and Rauch, 2012). Sakai et al. (2011) found that functional connections of the orbitofrontal cortex, medial prefrontal cortex, DLPFC and ventral striatum were significantly increased in patients with OCD, but there was no significant correlation between symptom severity and connection strength. D’Urso et al. (2016b) reported that patients received cathode stimulation over the left DLPFC showed significant improvement in OCD symptoms.

Generalized anxiety disorder is characterized by persistent unspecified nervousness, excessive anxiety and worry about everyday life events (Locke et al., 2015; Stein et al., 2017). Previous studies have shown that brain regions related to rumination and introspection in GAD patients were overactivated (Locke et al., 2015). Patients also showed autonomic nervous dysfunction, vagus-mediated decreased heart rate variability, and neurostructural abnormalities in the rostral ACC, left medial orbitofrontal cortex, and right isthmic cingulate gyrus (Etkin and Wager, 2007; Carnevali et al., 2019). Neuroplasticity in prefrontal and limbic regions is also altered in patients with a variety of subtypes of anxiety disorders (Ironside et al., 2019). Vicario et al. (2019) reviewed the using of non-invasive brain stimulation techniques for the treatment of anxiety previously. A study showed that stimulation of the left DLPFC with 2 mA tDCS significantly improved physical stress symptoms in patients, however, there was no significant improvement in major psychological symptoms, such as anxiety, tension, emotion, or depression (de Lima et al., 2019). In another case report, a total of 15 sessions of 2 mA cathode tDCS stimulation improved anxiety symptoms in patients with GAD (Shiozawa et al., 2014).

Social anxiety disorder is an anxiety disorder characterized by extreme fear in getting involved in social interactions. Studies have shown that patients with SAD have attentional bias brought by social threats, and the attentional bias will increase the anxiety of patients with SAD (Klosowska et al., 2015). Anode tDCS of the left DLPFC significantly reduced attentional bias compared to the sham stimulation (Heeren et al., 2017). In addition, a single dose of 1 mA of tDCS reduced pain anxiety caused by burns (Hosseini Amiri et al., 2016), and improved anxiety symptoms caused by major depression (Nishida et al., 2019). Although there are only a few studies on the tDCS treatment of anxiety, these findings indicate that this technique can be an effective therapeutic option. We have summarized some of the published studies in Table 5.

**TABLE 5:** Effects of tDCS on OCD and anxiety.

References	Disease	Electrode montage		Electrode size (cm^2^)	Current intensity (mA)	Stimulation duration (min)	Stimulation sessions	Total sessions	Key findings
		
		Anode (+)	Cathode (−)						
Narayanaswamy et al., 2015	OCD	Fz2	Right supra-orbital area	35	2	20	2/day × 10 days	20	Clinical improvement, enhanced pre-SMA/SMA activation
D’Urso et al., 2016a	OCD	Presupplementary motor area (pre-SMA)	Right deltoid	35	2	20	1/day × 20 days	20	Improved OCD symptoms
Shiozawa et al., 2014	OCD	Contralateral deltoid	Right DLPFC	25	2	20	1/day, 3 weeks	15	Improved anxiety symptoms
Volpato et al., 2013	OCD	Posterior neck-base	Left DLPFC (F3)	35	2	20	1/day, 10 days	10	Improved depression and anxiety, reduced interhemispheric imbalance
Ahmadizadeh et al., 2019	PTSD	DLPFC (F3)	Right DLPFC	35	2	20	1/day, 2 weeks	10	Reduced PTSD symptoms, hyper-arousal and negative alterations in cognition and mood sub-symptoms as well as depressive and anxiety symptoms
Jafari et al., 2021	SAD	DLPFC (F3)	Medial PFC (Fpz)	35	1/2	20	2/day × 5 days	10	Reduced fear/avoidance symptoms, worries and improved emotion regulation
de Lima et al., 2019	GAD	DLPFC (F3)	Contralateral supraorbital area (Fp2)	35	2	20	1/day, week	5	Improved in physical symptoms significantly, but no improvements in anxiety, mood symptoms of stress, affectivity, or depression
Heeren et al., 2017	SAD	DLPFC (F3)	Vertically at the ipsilateral arm	35	2	25	1/day	1	Decreased attentional bias

## Summary and Outlook

In recent years, tDCS is increasingly being studied for the therapeutic potential in neurological and psychiatric disorders. DLPFC is involved in many higher brain functions such as working memory, decision making, impulsivity, attention, etc. DLPFC also plays an important role in cognition and emotion. These brain functions were often disrupted in neurological and psychiatric diseases. Thus, modulation of the activity of DLPFC is a major strategy in treatment of these diseases. Although the neural mechanisms of tDCS is still not quite clear. It is believed that anodal stimulation increases brain activity while cathodal stimulation inhibits brain activity. One of the major problems of tDCS treatment of neuropsychiatric diseases is that each study used slightly different stimulation parameters. For instance, the current intensities were from 1 to 2 mA, tDCS sessions were from one session to more than 20 sessions. The tDCS frequency varies from twice daily to once every other day. Thus, it’s not appropriate to compare the current results directly side by side. Future studies will need to investigate the effects of tDCS using the different parameters in the same study or the same parameters in different studies. Nevertheless, this review demonstrates clearly that tDCS of DLPFC has a great potential to treat neuropsychiatric disorders.

## Author Contributions

CL and ZM discussed and initiated the review topic and edited the manuscript substantially. QL drafted the manuscript. All authors interpreted the results together, revised the manuscript critically, and contributed to the article and approved the submitted version.

## Conflict of Interest

The authors declare that the research was conducted in the absence of any commercial or financial relationships that could be construed as a potential conflict of interest.

## Publisher’s Note

All claims expressed in this article are solely those of the authors and do not necessarily represent those of their affiliated organizations, or those of the publisher, the editors and the reviewers. Any product that may be evaluated in this article, or claim that may be made by its manufacturer, is not guaranteed or endorsed by the publisher.

## References

[B1] Ahmadizadeh M. J. Rezaei M. Fitzgerald P. B. (2019). Transcranial direct current stimulation (tDCS) for post-traumatic stress disorder (PTSD): a randomized, double-blinded, controlled trial. *Brain Res. Bull.* 153 273–278. 10.1016/j.brainresbull.2019.09.011 31560945

[B2] Alghamdi F. Alhussien A. Alohali M. Alatawi A. Almusned T. Fecteau S. (2019). Effect of transcranial direct current stimulation on the number of smoked cigarettes in tobacco smokers. *PLoS One* 14:e0212312. 10.1371/journal.pone.0212312 30763404 PMC6375608

[B3] Allenby C. Falcone M. Bernardo L. Wileyto E. P. Rostain A. Ramsay J. R. (2018). Transcranial direct current brain stimulation decreases impulsivity in ADHD. *Brain Stimul.* 11 974–981. 10.1016/j.brs.2018.04.016 29885858 PMC6109423

[B4] Aparicio L. V. M. Rosa V. Razza L. M. Sampaio-Junior B. Borrione L. Valiengo L. (2019). Transcranial direct current stimulation (tDCS) for preventing major depressive disorder relapse: results of a 6-month follow-up. *Depress. Anxiety* 36 262–268. 10.1002/da.22878 30637889

[B5] Ayache S. S. Palm U. Chalah M. A. Al-Ani T. Brignol A. Abdellaoui M. (2016). Prefrontal tDCS decreases pain in patients with multiple sclerosis. *Front. Neurosci.* 10:147. 10.3389/fnins.2016.00147 27092048 PMC4824778

[B6] Barbey A. K. Koenigs M. Grafman J. (2013). Dorsolateral prefrontal contributions to human working memory. *Cortex* 49 1195–1205. 10.1016/j.cortex.2012.05.022 22789779 PMC3495093

[B7] Barros Galvao S. C. Borba Costa dos Santos R. Borba dos Santos P. Cabral M. E. Monte-Silva K. (2014). Efficacy of coupling repetitive transcranial magnetic stimulation and physical therapy to reduce upper-limb spasticity in patients with stroke: a randomized controlled trial. *Arch. Phys. Med. Rehabil.* 95 222–229. 10.1016/j.apmr.2013.10.023 24239881

[B8] Batista E. K. Klauss J. Fregni F. Nitsche M. A. Nakamura-Palacios E. M. (2015). A randomized placebo-controlled trial of targeted prefrontal cortex modulation with bilateral tDCS in patients with crack-cocaine dependence. *Int. J. Neuropsychopharmacol.* 18:pyv066. 10.1093/ijnp/pyv066 26065432 PMC4675977

[B9] Bennabi D. Nicolier M. Monnin J. Tio G. Pazart L. Vandel P. (2015). Pilot study of feasibility of the effect of treatment with tDCS in patients suffering from treatment-resistant depression treated with escitalopram. *Clin. Neurophysiol.* 126 1185–1189. 10.1016/j.clinph.2014.09.026 25454337

[B10] Berke J. D. Hyman S. E. (2000). Addiction, dopamine, and the molecular mechanisms of memory. *Neuron* 25 515–532. 10.1016/s0896-6273(00)81056-9 10774721

[B11] Bidet-Caulet A. Buchanan K. G. Viswanath H. Black J. Scabini D. Bonnet-Brilhault F. (2015). Impaired facilitatory mechanisms of auditory attention after damage of the lateral prefrontal cortex. *Cereb. Cortex* 25 4126–4134. 10.1093/cercor/bhu131 24925773 PMC4626830

[B12] Bikson M. Inoue M. Akiyama H. Deans J. K. Fox J. E. Miyakawa H. (2004). Effects of uniform extracellular DC electric fields on excitability in rat hippocampal slices in vitro. *J. Physiol.* 557(Pt 1) 175–190. 10.1113/jphysiol.2003.055772 14978199 PMC1665051

[B13] Blair C. Razza R. P. (2007). Relating effortful control, executive function, and false belief understanding to emerging math and literacy ability in kindergarten. *Child. Dev.* 78 647–663. 10.1111/j.1467-8624.2007.01019.x 17381795

[B14] Blumberger D. M. Tran L. C. Fitzgerald P. B. Hoy K. E. Daskalakis Z. J. (2012). A randomized double-blind sham-controlled study of transcranial direct current stimulation for treatment-resistant major depression. *Front. Psychiatry* 3:74. 10.3389/fpsyt.2012.00074 22912618 PMC3421236

[B15] Boggio P. S. Liguori P. Sultani N. Rezende L. Fecteau S. Fregni F. (2009). Cumulative priming effects of cortical stimulation on smoking cue-induced craving. *Neurosci. Lett.* 463 82–86. 10.1016/j.neulet.2009.07.041 19619607

[B16] Boggio P. S. Rigonatti S. P. Ribeiro R. B. Myczkowski M. L. Nitsche M. A. Pascual-Leone A. (2008a). A randomized, double-blind clinical trial on the efficacy of cortical direct current stimulation for the treatment of major depression. *Int. J. Neuropsychopharmacol.* 11 249–254. 10.1017/S1461145707007833 17559710 PMC3372849

[B17] Boggio P. S. Sultani N. Fecteau S. Merabet L. Mecca T. Pascual-Leone A. (2008b). Prefrontal cortex modulation using transcranial DC stimulation reduces alcohol craving: a double-blind, sham-controlled study. *Drug Alcohol Depend.* 92 55–60. 10.1016/j.drugalcdep.2007.06.011 17640830

[B18] Boning J. (2009). Addiction memory as a specific, individually learned memory imprint. *Pharmacopsychiatry* 42 (Suppl. 1) S66–S68. 10.1055/s-0029-1216357 19434557

[B19] Bose A. Shivakumar V. Narayanaswamy J. C. Nawani H. Subramaniam A. Agarwal S. M. (2014). Insight facilitation with add-on tDCS in schizophrenia. *Schizophr. Res.* 156 63–65. 10.1016/j.schres.2014.03.029 24767881

[B20] Bose A. Sowmya S. Shenoy S. Agarwal S. M. Chhabra H. Narayanaswamy J. C. (2015). Clinical utility of attentional salience in treatment of auditory verbal hallucinations in schizophrenia using transcranial direct current stimulation (tDCS). *Schizophr. Res.* 164 279–280. 10.1016/j.schres.2015.01.040 25691272

[B21] Brunelin J. Hasan A. Haesebaert F. Nitsche M. A. Poulet E. (2015). Nicotine smoking prevents the effects of frontotemporal transcranial direct current stimulation (tDCS) in hallucinating patients with schizophrenia. *Brain Stimul.* 8 1225–1227. 10.1016/j.brs.2015.08.002 26316227

[B22] Brunelin J. Mondino M. Gassab L. Haesebaert F. Gaha L. Suaud-Chagny M. F. (2012a). Examining transcranial direct-current stimulation (tDCS) as a treatment for hallucinations in schizophrenia. *Am. J. Psychiatry* 169 719–724. 10.1176/appi.ajp.2012.11071091 22581236

[B23] Brunelin J. Mondino M. Haesebaert F. Saoud M. Suaud-Chagny M. F. Poulet E. (2012b). Efficacy and safety of bifocal tDCS as an interventional treatment for refractory schizophrenia. *Brain Stimul.* 5 431–432. 10.1016/j.brs.2011.03.010 22037120

[B24] Brunoni A. R. Boggio P. S. De Raedt R. Bensenor I. M. Lotufo P. A. Namur V. (2014). Cognitive control therapy and transcranial direct current stimulation for depression: a randomized, double-blinded, controlled trial. *J. Affect. Disord.* 162 43–49. 10.1016/j.jad.2014.03.026 24767004

[B25] Brunoni A. R. Ferrucci R. Bortolomasi M. Scelzo E. Boggio P. S. Fregni F. (2013a). Interactions between transcranial direct current stimulation (tDCS) and pharmacological interventions in the major depressive episode: findings from a naturalistic study. *Eur. Psychiatry* 28 356–361. 10.1016/j.eurpsy.2012.09.001 23182847

[B26] Brunoni A. R. Valiengo L. Baccaro A. Zanao T. A. de Oliveira J. F. Goulart A. (2013b). The sertraline vs. electrical current therapy for treating depression clinical study: results from a factorial, randomized, controlled trial. *JAMA Psychiatry* 70 383–391. 10.1001/2013.jamapsychiatry.32 23389323

[B27] Brunoni A. R. Ferrucci R. Bortolomasi M. Vergari M. Tadini L. Boggio P. S. (2011). Transcranial direct current stimulation (tDCS) in unipolar vs. bipolar depressive disorder. *Prog. Neuropsychopharmacol. Biol. Psychiatry* 35 96–101. 10.1016/j.pnpbp.2010.09.010 20854868

[B28] Brunoni A. R. Moffa A. H. Sampaio-Junior B. Borrione L. Moreno M. L. Fernandes R. A. (2017). Trial of electrical direct-current therapy versus escitalopram for depression. *N. Engl. J. Med.* 376 2523–2533. 10.1056/NEJMoa1612999 28657871

[B29] Buhle J. T. Silvers J. A. Wager T. D. Lopez R. Onyemekwu C. Kober H. (2014). Cognitive reappraisal of emotion: a meta-analysis of human neuroimaging studies. *Cereb. Cortex* 24 2981–2990. 10.1093/cercor/bht154 23765157 PMC4193464

[B30] Cachoeira C. T. Leffa D. T. Mittelstadt S. D. Mendes L. S. T. Brunoni A. R. Pinto J. V. (2017). Positive effects of transcranial direct current stimulation in adult patients with attention-deficit/hyperactivity disorder – a pilot randomized controlled study. *Psychiatry Res.* 247 28–32. 10.1016/j.psychres.2016.11.009 27863315

[B31] Carnevali L. Mancini M. Koenig J. Makovac E. Watson D. R. Meeten F. (2019). Cortical morphometric predictors of autonomic dysfunction in generalized anxiety disorder. *Auton. Neurosci.* 217 41–48. 10.1016/j.autneu.2019.01.001 30704974

[B32] Carpenter W. T. Blanchard J. J. Kirkpatrick B. (2016). New standards for negative symptom assessment. *Schizophr. Bull.* 42 1–3. 10.1093/schbul/sbv160 26615186 PMC4681567

[B33] Chang C. C. Kao Y. C. Chao C. Y. Chang H. A. (2019). Enhancement of cognitive insight and higher-order neurocognitive function by fronto-temporal transcranial direct current stimulation (tDCS) in patients with schizophrenia. *Schizophr. Res.* 208 430–438. 10.1016/j.schres.2018.12.052 30635256

[B34] Chang C. C. Kao Y. C. Chao C. Y. Tzeng N. S. Chang H. A. (2020). Examining bi-anodal transcranial direct current stimulation (tDCS) over bilateral dorsolateral prefrontal cortex coupled with bilateral extracephalic references as a treatment for negative symptoms in non-acute schizophrenia patients: a randomized, double-blind, sham-controlled trial. *Prog. Neuropsychopharmacol. Biol. Psychiatry.* 96:109715. 10.1016/j.pnpbp.2019.109715 31362034

[B35] Christakou A. Murphy C. M. Chantiluke K. Cubillo A. I. Smith A. B. Giampietro V. (2013). Disorder-specific functional abnormalities during sustained attention in youth with attention deficit hyperactivity disorder (ADHD) and with autism. *Mol. Psychiatry* 18 236–244. 10.1038/mp.2011.185 22290121 PMC3554878

[B36] Cosmo C. Baptista A. F. de Araujo A. N. do Rosario R. S. Miranda J. G. Montoya P. (2015). A randomized, double-blind, sham-controlled trial of transcranial direct current stimulation in attention-deficit/hyperactivity disorder. *PLoS One* 10:e0135371. 10.1371/journal.pone.0135371 26267861 PMC4534404

[B37] Cubillo A. Halari R. Ecker C. Giampietro V. Taylor E. Rubia K. (2010). Reduced activation and inter-regional functional connectivity of fronto-striatal networks in adults with childhood attention-deficit hyperactivity disorder (ADHD) and persisting symptoms during tasks of motor inhibition and cognitive switching. *J. Psychiatr. Res.* 44 629–639. 10.1016/j.jpsychires.2009.11.016 20060129

[B38] Cubillo A. Halari R. Smith A. Taylor E. Rubia K. (2012). A review of fronto-striatal and fronto-cortical brain abnormalities in children and adults with attention deficit hyperactivity disorder (ADHD) and new evidence for dysfunction in adults with ADHD during motivation and attention. *Cortex* 48 194–215. 10.1016/j.cortex.2011.04.007 21575934

[B39] da Silva M. C. Conti C. L. Klauss J. Alves L. G. do Nascimento Cavalcante H. M. Fregni F. (2013). Behavioral effects of transcranial direct current stimulation (tDCS) induced dorsolateral prefrontal cortex plasticity in alcohol dependence. *J. Physiol. Paris* 107 493–502. 10.1016/j.jphysparis.2013.07.003 23891741

[B40] Dalong G. Jiyuan L. Ying Z. Lei Z. Yanhong H. Yongcong S. (2020). Transcranial direct current stimulation reconstructs diminished thalamocortical connectivity during prolonged resting wakefulness: a resting-state fMRI pilot study. *Brain Imaging Behav.* 14 278–288. 10.1007/s11682-018-9979-9 30430411

[B41] de Lima A. L. Braga F. M. A. da Costa R. M. M. Gomes E. P. Brunoni A. R. Pegado R. (2019). Transcranial direct current stimulation for the treatment of generalized anxiety disorder: a randomized clinical trial. *J. Affect. Disord.* 259 31–37. 10.1016/j.jad.2019.08.020 31437698

[B42] Del Casale A. Kotzalidis G. D. Rapinesi C. Serata D. Ambrosi E. Simonetti A. (2011). Functional neuroimaging in obsessive-compulsive disorder. *Neuropsychobiology* 64 61–85. 10.1159/000325223 21701225

[B43] Dell’Osso B. Zanoni S. Ferrucci R. Vergari M. Castellano F. D’Urso N. (2012). Transcranial direct current stimulation for the outpatient treatment of poor-responder depressed patients. *Eur. Psychiatry* 27 513–517. 10.1016/j.eurpsy.2011.02.008 21621982

[B44] den Uyl T. E. Gladwin T. E. Rinck M. Lindenmeyer J. Wiers R. W. (2017). A clinical trial with combined transcranial direct current stimulation and alcohol approach bias retraining. *Addict. Biol.* 22 1632–1640. 10.1111/adb.12463 27790791

[B45] den Uyl T. E. Gladwin T. E. Wiers R. W. (2015). Transcranial direct current stimulation, implicit alcohol associations and craving. *Biol. Psychol.* 105 37–42. 10.1016/j.biopsycho.2014.12.004 25541515

[B46] den Uyl T. E. Gladwin T. E. Wiers R. W. (2016). Electrophysiological and behavioral effects of combined transcranial direct current stimulation and alcohol approach bias retraining in hazardous drinkers. *Alcohol Clin. Exp. Res.* 40 2124–2133. 10.1111/acer.13171 27558788

[B47] Dubreuil-Vall L. Gomez-Bernal F. Villegas A. C. Cirillo P. Surman C. Ruffini G. (2021). Transcranial direct current stimulation to the left dorsolateral prefrontal cortex improves cognitive control in patients with attention-deficit/hyperactivity disorder: a randomized behavioral and neurophysiological study. *Biol. Psychiatry Cogn. Neurosci. Neuroimaging* 6 439–448. 10.1016/j.bpsc.2020.11.006 33549516 PMC8103824

[B48] D’Urso G. Brunoni A. R. Anastasia A. Micillo M. de Bartolomeis A. Mantovani A. (2016a). Polarity-dependent effects of transcranial direct current stimulation in obsessive-compulsive disorder. *Neurocase* 22 60–64. 10.1080/13554794.2015.1045522 25971992

[B49] D’Urso G. Brunoni A. R. Mazzaferro M. P. Anastasia A. de Bartolomeis A. Mantovani A. (2016b). Transcranial direct current stimulation for obsessive-compulsive disorder: a randomized, controlled, partial crossover trial. *Depress. Anxiety* 33 1132–1140. 10.1002/da.22578 27802585

[B50] Durston S. Tottenham N. T. Thomas K. M. Davidson M. C. Eigsti I. M. Yang Y. (2003). Differential patterns of striatal activation in young children with and without ADHD. *Biol. Psychiatry* 53 871–878. 10.1016/s0006-3223(02)01904-2 12742674

[B51] Etkin A. Wager T. D. (2007). Functional neuroimaging of anxiety: a meta-analysis of emotional processing in PTSD, social anxiety disorder, and specific phobia. *Am. J. Psychiatry* 164 1476–1488. 10.1176/appi.ajp.2007.07030504 17898336 PMC3318959

[B52] Falcone M. Bernardo L. Ashare R. L. Hamilton R. Faseyitan O. McKee S. A. (2016). Transcranial direct current brain stimulation increases ability to resist smoking. *Brain Stimul.* 9 191–196. 10.1016/j.brs.2015.10.004 26572280 PMC4789149

[B53] Fecteau S. Agosta S. Hone-Blanchet A. Fregni F. Boggio P. Ciraulo D. (2014). Modulation of smoking and decision-making behaviors with transcranial direct current stimulation in tobacco smokers: a preliminary study. *Drug Alcohol Depend.* 140 78–84. 10.1016/j.drugalcdep.2014.03.036 24814566 PMC4242508

[B54] Fernandez-Serrano M. J. Perez-Garcia M. Perales J. C. Verdejo-Garcia A. (2010). Prevalence of executive dysfunction in cocaine, heroin and alcohol users enrolled in therapeutic communities. *Eur. J. Pharmacol.* 626 104–112. 10.1016/j.ejphar.2009.10.019 19836375

[B55] Fineberg N. A. Chamberlain S. R. Hollander E. Boulougouris V. Robbins T. W. (2011). Translational approaches to obsessive-compulsive disorder: from animal models to clinical treatment. *Br. J. Pharmacol.* 164 1044–1061. 10.1111/j.1476-5381.2011.01422.x 21486280 PMC3229751

[B56] Fitzgerald P. B. McQueen S. Daskalakis Z. J. Hoy K. E. (2014). A negative pilot study of daily bimodal transcranial direct current stimulation in schizophrenia. *Brain Stimul.* 7 813–816. 10.1016/j.brs.2014.08.002 25442152

[B57] Fitzpatrick S. Gilbert S. Serpell L. (2013). Systematic review: are overweight and obese individuals impaired on behavioural tasks of executive functioning? *Neuropsychol. Rev.* 23 138–156. 10.1007/s11065-013-9224-7 23381140

[B58] Francis J. T. Gluckman B. J. Schiff S. J. (2003). Sensitivity of neurons to weak electric fields. *J. Neurosci.* 23 7255–7261. 10.1523/JNEUROSCI.23-19-07255.2003 12917358 PMC6740448

[B59] Frank D. W. Dewitt M. Hudgens-Haney M. Schaeffer D. J. Ball B. H. Schwarz N. F. (2014). Emotion regulation: quantitative meta-analysis of functional activation and deactivation. *Neurosci. Biobehav. Rev.* 45 202–211. 10.1016/j.neubiorev.2014.06.010 24984244

[B60] Fregni F. Liguori P. Fecteau S. Nitsche M. A. Pascual-Leone A. Boggio P. S. (2008a). Cortical stimulation of the prefrontal cortex with transcranial direct current stimulation reduces cue-provoked smoking craving: a randomized, sham-controlled study. *J. Clin. Psychiatry* 69 32–40. 10.4088/jcp.v69n0105 18312035

[B61] Fregni F. Orsati F. Pedrosa W. Fecteau S. Tome F. A. Nitsche M. A. (2008b). Transcranial direct current stimulation of the prefrontal cortex modulates the desire for specific foods. *Appetite* 51 34–41. 10.1016/j.appet.2007.09.016 18243412 PMC3541023

[B62] Fritsch B. Reis J. Martinowich K. Schambra H. M. Ji Y. Cohen L. G. (2010). Direct current stimulation promotes BDNF-dependent synaptic plasticity: potential implications for motor learning. *Neuron* 66 198–204. 10.1016/j.neuron.2010.03.035 20434997 PMC2864780

[B63] Frohlich F. Burrello T. N. Mellin J. M. Cordle A. L. Lustenberger C. M. Gilmore J. H. (2016). Exploratory study of once-daily transcranial direct current stimulation (tDCS) as a treatment for auditory hallucinations in schizophrenia. *Eur. Psychiatry* 33 54–60. 10.1016/j.eurpsy.2015.11.005 26866874

[B64] Ghorbani Behnam S. Mousavi S. A. Emamian M. H. (2019). The effects of transcranial direct current stimulation compared to standard bupropion for the treatment of tobacco dependence: a randomized sham-controlled trial. *Eur. Psychiatry* 60 41–48. 10.1016/j.eurpsy.2019.04.010 31100611

[B65] Glasser M. F. Coalson T. S. Robinson E. C. Hacker C. D. Harwell J. Yacoub E. (2016). A multi-modal parcellation of human cerebral cortex. *Nature* 536 171–178. 10.1038/nature18933 27437579 PMC4990127

[B66] Gogler N. Willacker L. Funk J. Strube W. Langgartner S. Napiorkowski N. (2017). Single-session transcranial direct current stimulation induces enduring enhancement of visual processing speed in patients with major depression. *Eur. Arch. Psychiatry Clin. Neurosci.* 267 671–686. 10.1007/s00406-016-0761-y 28039551

[B67] Goldstein R. Z. Volkow N. D. (2002). Drug addiction and its underlying neurobiological basis: neuroimaging evidence for the involvement of the frontal cortex. *Am. J. Psychiatry* 159 1642–1652. 10.1176/appi.ajp.159.10.1642 12359667 PMC1201373

[B68] Gordon P. C. Valiengo L. de Paula V. J. R. Galhardoni R. Ziemann U. de Andrade D. C. (2019). Changes in motor cortical excitability in schizophrenia following transcranial direct current stimulation. *Prog. Neuropsychopharmacol. Biol. Psychiatry* 90 43–48. 10.1016/j.pnpbp.2018.11.004 30423420

[B69] Gorini A. Lucchiari C. Russell-Edu W. Pravettoni G. (2014). Modulation of risky choices in recently abstinent dependent cocaine users: a transcranial direct-current stimulation study. *Front. Hum. Neurosci.* 8:661. 10.3389/fnhum.2014.00661 25221496 PMC4145470

[B70] Hart H. Radua J. Nakao T. Mataix-Cols D. Rubia K. (2013). Meta-analysis of functional magnetic resonance imaging studies of inhibition and attention in attention-deficit/hyperactivity disorder: exploring task-specific, stimulant medication, and age effects. *JAMA Psychiatry* 70 185–198. 10.1001/jamapsychiatry.2013.277 23247506

[B71] Hecht D. (2010). Depression and the hyperactive right-hemisphere. *Neurosci. Res.* 68 77–87. 10.1016/j.neures.2010.06.013 20603163

[B72] Heeren A. Billieux J. Philippot P. De Raedt R. Baeken C. de Timary P. (2017). Impact of transcranial direct current stimulation on attentional bias for threat: a proof-of-concept study among individuals with social anxiety disorder. *Soc. Cogn. Affect. Neurosci.* 12 251–260. 10.1093/scan/nsw119 27531388 PMC5390730

[B73] Hill A. T. Fitzgerald P. B. Hoy K. E. (2016). Effects of anodal transcranial direct current stimulation on working memory: a systematic review and meta-analysis of findings from healthy and neuropsychiatric populations. *Brain Stimul.* 9 197–208. 10.1016/j.brs.2015.10.006 26597929

[B74] Holla B. Biswal J. Ramesh V. Shivakumar V. Bharath R. D. Benegal V. (2020). Effect of prefrontal tDCS on resting brain fMRI graph measures in alcohol use disorders: a randomized, double-blind, sham-controlled study. *Prog. Neuropsychopharmacol. Biol. Psychiatry* 102:109950. 10.1016/j.pnpbp.2020.109950 32339664

[B75] Homan P. Kindler J. Federspiel A. Flury R. Hubl D. Hauf M. (2011). Muting the voice: a case of arterial spin labeling-monitored transcranial direct current stimulation treatment of auditory verbal hallucinations. *Am. J. Psychiatry* 168 853–854. 10.1176/appi.ajp.2011.11030496 21813497

[B76] Hosseini Amiri M. Tavousi S. H. Mazlom S. R. Manzari Z. S. (2016). Effect of transcranial direct current stimulation on pain anxiety during burn wound care. *Burns* 42 872–876. 10.1016/j.burns.2016.01.006 26827187

[B77] Hoy K. E. Arnold S. L. Emonson M. R. Daskalakis Z. J. Fitzgerald P. B. (2014). An investigation into the effects of tDCS dose on cognitive performance over time in patients with schizophrenia. *Schizophr. Res.* 155 96–100. 10.1016/j.schres.2014.03.006 24703529

[B78] Hyman S. E. Malenka R. C. (2001). Addiction and the brain: the neurobiology of compulsion and its persistence. *Nat. Rev. Neurosci.* 2 695–703. 10.1038/35094560 11584307

[B79] Hyman S. E. Malenka R. C. Nestler E. J. (2006). Neural mechanisms of addiction: the role of reward-related learning and memory. *Annu. Rev. Neurosci.* 29 565–598. 10.1146/annurev.neuro.29.051605.113009 16776597

[B80] Ironside M. Browning M. Ansari T. L. Harvey C. J. Sekyi-Djan M. N. Bishop S. J. (2019). Effect of prefrontal cortex stimulation on regulation of amygdala response to threat in individuals with trait anxiety: a randomized clinical trial. *JAMA Psychiatry* 76 71–78. 10.1001/jamapsychiatry.2018.2172 30347011 PMC6583758

[B81] Jacks S. Kalivas B. Mittendorf A. Kindt C. Short E. B. (2014). Transcranial direct-current stimulation as an adjunct to electroconvulsive therapy and clozapine for refractory psychosis. *Prim. Care Companion CNS Disord.* 16:PCC.14l01635. 10.4088/PCC.14l01635 25317373 PMC4195645

[B82] Jafari E. Alizadehgoradel J. Pourmohseni Koluri F. Nikoozadehkordmirza E. Refahi M. Taherifard M. (2021). Intensified electrical stimulation targeting lateral and medial prefrontal cortices for the treatment of social anxiety disorder: a randomized, double-blind, parallel-group, dose-comparison study. *Brain Stimul.* 14 974–986. 10.1016/j.brs.2021.06.005 34167918

[B83] Jeon D. W. Jung D. U. Kim S. J. Shim J. C. Moon J. J. Seo Y. S. (2018). Adjunct transcranial direct current stimulation improves cognitive function in patients with schizophrenia: a double-blind 12-week study. *Schizophr. Res.* 197 378–385. 10.1016/j.schres.2017.12.009 30955702

[B84] Klauss J. Anders Q. S. Felippe L. V. Ferreira L. V. B. Cruz M. A. Nitsche M. A. (2018a). Lack of effects of extended sessions of transcranial direct current stimulation (tDCS) over dorsolateral prefrontal cortex on craving and relapses in crack-cocaine users. *Front. Pharmacol.* 9:1198. 10.3389/fphar.2018.01198 30405414 PMC6206545

[B85] Klauss J. Anders Q. S. Felippe L. V. Nitsche M. A. Nakamura-Palacios E. M. (2018b). Multiple sessions of transcranial direct current stimulation (tDCS) reduced craving and relapses for alcohol use: a randomized placebo-controlled trial in alcohol use disorder. *Front. Pharmacol.* 9:716. 10.3389/fphar.2018.00716 30018558 PMC6037838

[B86] Klosowska J. Blaut A. Paulewicz B. (2015). [Attentional bias training in reducing symptoms of anxiety]. *Psychiatr. Pol.* 49 57–66. 10.12740/PP/27628 25844410

[B87] Koenigs M. Grafman J. (2009). The functional neuroanatomy of depression: distinct roles for ventromedial and dorsolateral prefrontal cortex. *Behav. Brain Res.* 201 239–243. 10.1016/j.bbr.2009.03.004 19428640 PMC2680780

[B88] Kroczek A. M. Haussinger F. B. Rohe T. Schneider S. Plewnia C. Batra A. (2016). Effects of transcranial direct current stimulation on craving, heart-rate variability and prefrontal hemodynamics during smoking cue exposure. *Drug Alcohol Depend.* 168 123–127. 10.1016/j.drugalcdep.2016.09.006 27639130

[B89] Kumar S. Batist J. Ghazala Z. Zomorrodi R. M. Brooks H. Goodman M. (2020). Effects of bilateral transcranial direct current stimulation on working memory and global cognition in older patients with remitted major depression: a pilot randomized clinical trial. *Int. J. Geriatr. Psychiatry* 35 1233–1242. 10.1002/gps.5361 32525222

[B90] Liebetanz D. Nitsche M. A. Tergau F. Paulus W. (2002). Pharmacological approach to the mechanisms of transcranial DC-stimulation-induced after-effects of human motor cortex excitability. *Brain* 125(Pt 10) 2238–2247. 10.1093/brain/awf238 12244081

[B91] Lin Y. Y. Chang C. C. Huang C. C. Tzeng N. S. Kao Y. C. Chang H. A. (2021). Efficacy and neurophysiological predictors of treatment response of adjunct bifrontal transcranial direct current stimulation (tDCS) in treating unipolar and bipolar depression. *J. Affect. Disord.* 280(Pt A) 295–304. 10.1016/j.jad.2020.11.030 33221715

[B92] Locke A. B. Kirst N. Shultz C. G. (2015). Diagnosis and management of generalized anxiety disorder and panic disorder in adults. *Am. Fam. Phys.* 91 617–624. 25955736

[B93] Loo C. K. Alonzo A. Martin D. Mitchell P. B. Galvez V. Sachdev P. (2012). Transcranial direct current stimulation for depression: 3-week, randomised, sham-controlled trial. *Br. J. Psychiatry* 200 52–59. 10.1192/bjp.bp.111.097634 22215866

[B94] Loo C. K. Sachdev P. Martin D. Pigot M. Alonzo A. Malhi G. S. (2010). A double-blind, sham-controlled trial of transcranial direct current stimulation for the treatment of depression. *Int. J. Neuropsychopharmacol.* 13 61–69. 10.1017/S1461145709990411 19671217

[B95] Marazziti D. Presta S. Baroni S. Silvestri S. Dell’Osso L. (2014). Behavioral addictions: a novel challenge for psychopharmacology. *CNS Spectr.* 19 486–495. 10.1017/S1092852913001041 24589040

[B96] Martin D. M. Alonzo A. Ho K. A. Player M. Mitchell P. B. Sachdev P. (2013). Continuation transcranial direct current stimulation for the prevention of relapse in major depression. *J. Affect. Disord.* 144 274–278. 10.1016/j.jad.2012.10.012 23146197

[B97] McDermott T. J. Wiesman A. I. Mills M. S. Spooner R. K. Coolidge N. M. Proskovec A. L. (2019). tDCS modulates behavioral performance and the neural oscillatory dynamics serving visual selective attention. *Hum. Brain Mapp.* 40 729–740. 10.1002/hbm.24405 30368974 PMC6328324

[B98] Milad M. R. Rauch S. L. (2012). Obsessive-compulsive disorder: beyond segregated cortico-striatal pathways. *Trends Cogn. Sci.* 16 43–51. 10.1016/j.tics.2011.11.003 22138231 PMC4955838

[B99] Mondino M. Haesebaert F. Poulet E. Suaud-Chagny M. F. Brunelin J. (2015). Fronto-temporal transcranial direct current stimulation (tDCS) reduces source-monitoring deficits and auditory hallucinations in patients with schizophrenia. *Schizophr. Res.* 161 515–516. 10.1016/j.schres.2014.10.054 25468175

[B100] Mondino M. Jardri R. Suaud-Chagny M. F. Saoud M. Poulet E. Brunelin J. (2016). Effects of fronto-temporal transcranial direct current stimulation on auditory verbal hallucinations and resting-state functional connectivity of the left temporo-parietal junction in patients with schizophrenia. *Schizophr. Bull.* 42 318–326. 10.1093/schbul/sbv114 26303936 PMC4753593

[B101] Mondino M. Luck D. Grot S. Januel D. Suaud-Chagny M. F. Poulet E. (2018). Effects of repeated transcranial direct current stimulation on smoking, craving and brain reactivity to smoking cues. *Sci. Rep.* 8:8724. 10.1038/s41598-018-27057-1 29880873 PMC5992174

[B102] Moreno M. L. Goerigk S. A. Bertola L. Suemoto C. K. Razza L. B. Moffa A. H. (2020). Cognitive changes after tDCS and escitalopram treatment in major depressive disorder: results from the placebo-controlled ELECT-TDCS trial. *J. Affect. Disord.* 263 344–352. 10.1016/j.jad.2019.12.009 31969264

[B103] Narayanaswamy J. C. Jose D. Chhabra H. Agarwal S. M. Shrinivasa B. Hegde A. (2015). Successful application of add-on transcranial direct current stimulation (tDCS) for treatment of SSRI resistant OCD. *Brain Stimul.* 8 655–657. 10.1016/j.brs.2014.12.003 25583654

[B104] Narayanaswamy J. C. Shivakumar V. Bose A. Agarwal S. M. Venkatasubramanian G. Gangadhar B. N. (2014). Sustained improvement of negative symptoms in schizophrenia with add-on tDCS: a case report. *Clin. Schizophr. Relat. Psychoses* 8 135–136. 10.3371/CSRP.JNVS.061314 24951718

[B105] Nawani H. Bose A. Agarwal S. M. Shivakumar V. Chhabra H. Subramaniam A. (2014a). Modulation of corollary discharge dysfunction in schizophrenia by tDCS: preliminary evidence. *Brain Stimul.* 7 486–488. 10.1016/j.brs.2014.01.003 24507573

[B106] Nawani H. Kalmady S. V. Bose A. Shivakumar V. Rakesh G. Subramaniam A. (2014b). Neural basis of tDCS effects on auditory verbal hallucinations in schizophrenia: a case report evidence for cortical neuroplasticity modulation. *J. ECT* 30 e2–e4. 10.1097/YCT.0b013e3182a35492 24080544

[B107] Nejati V. Salehinejad M. A. Nitsche M. A. Najian A. Javadi A. H. (2020). Transcranial direct current stimulation improves executive dysfunctions in ADHD: implications for inhibitory control, interference control, working memory, and cognitive flexibility. *J. Atten. Disord.* 24 1928–1943. 10.1177/1087054717730611 28938852

[B108] Nestler E. J. (2013). Cellular basis of memory for addiction. *Dialog. Clin. Neurosci.* 15 431–443. 10.31887/DCNS.2013.15.4/enestler 24459410 PMC3898681

[B109] Nishida K. Koshikawa Y. Morishima Y. Yoshimura M. Katsura K. Ueda S. (2019). Pre-stimulus brain activity is associated with state-anxiety changes during single-session transcranial direct current stimulation. *Front. Hum. Neurosci.* 13:266. 10.3389/fnhum.2019.00266 31440149 PMC6694795

[B110] Orlov N. D. Tracy D. K. Joyce D. Patel S. Rodzinka-Pasko J. Dolan H. (2017). Stimulating cognition in schizophrenia: a controlled pilot study of the effects of prefrontal transcranial direct current stimulation upon memory and learning. *Brain Stimul.* 10 560–566. 10.1016/j.brs.2016.12.013 28057452

[B111] Palm U. Keeser D. Blautzik J. Pogarell O. Ertl-Wagner B. Kupka M. J. (2013). Prefrontal transcranial direct current stimulation (tDCS) changes negative symptoms and functional connectivity MRI (fcMRI) in a single case of treatment-resistant schizophrenia. *Schizophr. Res.* 150 583–585. 10.1016/j.schres.2013.08.043 24060570

[B112] Palm U. Keeser D. Hasan A. Kupka M. J. Blautzik J. Sarubin N. (2016). Prefrontal transcranial direct current stimulation for treatment of schizophrenia with predominant negative symptoms: a double-blind, sham-controlled proof-of-concept study. *Schizophr. Bull.* 42 1253–1261. 10.1093/schbul/sbw041 27098066 PMC4988747

[B113] Palm U. Schiller C. Fintescu Z. Obermeier M. Keeser D. Reisinger E. (2012). Transcranial direct current stimulation in treatment resistant depression: a randomized double-blind, placebo-controlled study. *Brain Stimul.* 5 242–251. 10.1016/j.brs.2011.08.005 21962978

[B114] Pena-Gomez C. Vidal-Pineiro D. Clemente I. C. Pascual-Leone A. Bartres-Faz D. (2011). Down-regulation of negative emotional processing by transcranial direct current stimulation: effects of personality characteristics. *PLoS One* 6:e22812. 10.1371/journal.pone.0022812 21829522 PMC3146508

[B115] Philiastides M. G. Auksztulewicz R. Heekeren H. R. Blankenburg F. (2011). Causal role of dorsolateral prefrontal cortex in human perceptual decision making. *Curr. Biol.* 21 980–983. 10.1016/j.cub.2011.04.034 21620706

[B116] Pierce R. C. Kumaresan V. (2006). The mesolimbic dopamine system: the final common pathway for the reinforcing effect of drugs of abuse? *Neurosci. Biobehav. Rev.* 30 215–238. 10.1016/j.neubiorev.2005.04.016 16099045

[B117] Polanczyk G. V. Salum G. A. Sugaya L. S. Caye A. Rohde L. A. (2015). Annual research review: a meta-analysis of the worldwide prevalence of mental disorders in children and adolescents. *J. Child Psychol. Psychiatry* 56 345–365. 10.1111/jcpp.12381 25649325

[B118] Potenza M. N. (2014). Non-substance addictive behaviors in the context of DSM-5. *Addict. Behav.* 39 1–2. 10.1016/j.addbeh.2013.09.004 24119712 PMC3858502

[B119] Praharaj S. K. Behere R. V. Sharma P. S. (2015). Cathodal transcranial direct current stimulation over left temporoparietal area for treatment-refractory delusions and auditory hallucinations in schizophrenia: a case study. *J. ECT* 31 277–278. 10.1097/YCT.0000000000000237 25807343

[B120] Preller K. H. Wagner M. Sulzbach C. Hoenig K. Neubauer J. Franke P. E. (2013). Sustained incentive value of heroin-related cues in short- and long-term abstinent heroin users. *Eur. Neuropsychopharmacol.* 23 1270–1279. 10.1016/j.euroneuro.2012.11.007 23219936

[B121] Purpura D. P. McMurtry J. G. (1965). Intracellular activities and evoked potential changes during polarization of motor cortex. *J. Neurophysiol.* 28 166–185. 10.1152/jn.1965.28.1.166 14244793

[B122] Rahnev D. Nee D. E. Riddle J. Larson A. S. D’Esposito M. (2016). Causal evidence for frontal cortex organization for perceptual decision making. *Proc. Natl. Acad. Sci. U.S.A.* 113 6059–6064. 10.1073/pnas.1522551113 27162349 PMC4889369

[B123] Rakesh G. Shivakumar V. Subramaniam A. Nawani H. Amaresha A. C. Narayanaswamy J. C. (2013). Monotherapy with tDCS for schizophrenia: a case report. *Brain Stimul.* 6 708–709. 10.1016/j.brs.2013.01.007 23433875

[B124] Rigonatti S. P. Boggio P. S. Myczkowski M. L. Otta E. Fiquer J. T. Ribeiro R. B. (2008). Transcranial direct stimulation and fluoxetine for the treatment of depression. *Eur. Psychiatry.* 23 74–76. 10.1016/j.eurpsy.2007.09.006 18023968

[B125] Robbins T. W. Everitt B. J. (2002). Limbic-striatal memory systems and drug addiction. *Neurobiol. Learn. Mem.* 78 625–636. 10.1006/nlme.2002.4103 12559840

[B126] Rowland A. S. Skipper B. J. Umbach D. M. Rabiner D. L. Campbell R. A. Naftel A. J. (2015). The prevalence of ADHD in a population-based sample. *J. Atten. Disord.* 19 741–754. 10.1177/1087054713513799 24336124 PMC4058092

[B127] Rubi-Fessen I. Hartmann A. Huber W. Fimm B. Rommel T. Thiel A. (2015). Add-on effects of repetitive transcranial magnetic stimulation on subacute aphasia therapy: enhanced improvement of functional communication and basic linguistic skills. A randomized controlled study. *Arch. Phys. Med. Rehabil.* 96 1935–1944.e1932. 10.1016/j.apmr.2015.06.017 26189201

[B128] Sakai Y. Narumoto J. Nishida S. Nakamae T. Yamada K. Nishimura T. (2011). Corticostriatal functional connectivity in non-medicated patients with obsessive-compulsive disorder. *Eur. Psychiatry* 26 463–469. 10.1016/j.eurpsy.2010.09.005 21067900

[B129] Sampaio-Junior B. Tortella G. Borrione L. Moffa A. H. Machado-Vieira R. Cretaz E. (2018). Efficacy and safety of transcranial direct current stimulation as an add-on treatment for bipolar depression: a randomized clinical trial. *JAMA Psychiatry* 75 158–166. 10.1001/jamapsychiatry.2017.4040 29282470 PMC5838572

[B130] Schilling T. M. Bossert M. Konig M. Wirtz G. Weisbrod M. Aschenbrenner S. (2021). Acute effects of a single dose of 2 mA of anodal transcranial direct current stimulation over the left dorsolateral prefrontal cortex on executive functions in patients with schizophrenia-a randomized controlled trial. *PLoS One* 16:e0254695. 10.1371/journal.pone.0254695 34270620 PMC8284793

[B131] Shahani B. Russell W. R. (1969). Motor neurone disease. An abnormality of nerve metabolism. *J. Neurol. Neurosurg. Psychiatry* 32 1–5. 10.1136/jnnp.32.1.1 5774129 PMC493376

[B132] Sharafi E. Taghva A. Arbabi M. Dadarkhah A. Ghaderi J. (2019). Transcranial direct current stimulation for treatment-resistant major depression: a double-blind randomized sham-controlled trial. *Clin. EEG Neurosci.* 50 375–382. 10.1177/1550059419863209 31304775

[B133] Shenoy S. Bose A. Chhabra H. Dinakaran D. Agarwal S. M. Shivakumar V. (2015). Transcranial direct current stimulation (tDCS) for auditory verbal hallucinations in schizophrenia during pregnancy: a case report. *Brain Stimul.* 8 163–164. 10.1016/j.brs.2014.10.013 25468071

[B134] Shiozawa P. da Silva M. E. Cordeiro Q. Fregni F. Brunoni A. R. (2013). Transcranial direct current stimulation (tDCS) for catatonic schizophrenia: a case study. *Schizophr. Res.* 146 374–375. 10.1016/j.schres.2013.01.030 23434501

[B135] Shiozawa P. Gomes J. S. Ducos D. V. Akiba H. T. Dias A. M. Trevizol A. P. (2016). Effect of transcranial direct current stimulation (tDCS) over the prefrontal cortex combined with cognitive training for treating schizophrenia: a sham-controlled randomized clinical trial. *Trends Psychiatry Psychother.* 38 175–177. 10.1590/2237-6089-2015-0043 27737311

[B136] Shiozawa P. Leiva A. P. Castro C. D. da Silva M. E. Cordeiro Q. Fregni F. (2014). Transcranial direct current stimulation for generalized anxiety disorder: a case study. *Biol. Psychiatry* 75 e17–e18. 10.1016/j.biopsych.2013.07.014 23958182

[B137] Smith R. C. Boules S. Mattiuz S. Youssef M. Tobe R. H. Sershen H. (2015). Effects of transcranial direct current stimulation (tDCS) on cognition, symptoms, and smoking in schizophrenia: a randomized controlled study. *Schizophr. Res.* 168 260–266. 10.1016/j.schres.2015.06.011 26190299

[B138] Soff C. Sotnikova A. Christiansen H. Becker K. Siniatchkin M. (2017). Transcranial direct current stimulation improves clinical symptoms in adolescents with attention deficit hyperactivity disorder. *J. Neural. Transm (Vienna).* 124 133–144. 10.1007/s00702-016-1646-y 27853926

[B139] Stagg C. J. Best J. G. Stephenson M. C. O’Shea J. Wylezinska M. Kincses Z. T. (2009). Polarity-sensitive modulation of cortical neurotransmitters by transcranial stimulation. *J. Neurosci.* 29 5202–5206. 10.1523/JNEUROSCI.4432-08.2009 19386916 PMC6665468

[B140] Stein D. J. Scott K. M. de Jonge P. Kessler R. C. (2017). Epidemiology of anxiety disorders: from surveys to nosology and back. *Dialog. Clin. Neurosci.* 19 127–136. 10.31887/DCNS.2017.19.2/dstein 28867937 PMC5573557

[B141] Valiengo L. Goerigk S. Gordon P. C. Padberg F. Serpa M. H. Koebe S. (2020). Efficacy and safety of transcranial direct current stimulation for treating negative symptoms in schizophrenia: a randomized clinical trial. *JAMA Psychiatry* 77 121–129. 10.1001/jamapsychiatry.2019.3199 31617873 PMC6802484

[B142] Vaseghi B. Zoghi M. Jaberzadeh S. (2015). A meta-analysis of site-specific effects of cathodal transcranial direct current stimulation on sensory perception and pain. *PLoS One* 10:e0123873. 10.1371/journal.pone.0123873 25978673 PMC4433259

[B143] Vicario C. M. Salehinejad M. A. Felmingham K. Martino G. Nitsche M. A. (2019). A systematic review on the therapeutic effectiveness of non-invasive brain stimulation for the treatment of anxiety disorders. *Neurosci. Biobehav. Rev.* 96 219–231. 10.1016/j.neubiorev.2018.12.012 30543906

[B144] Vitor de Souza Brangioni M. C. Pereira D. A. Thibaut A. Fregni F. Brasil-Neto J. P. Boechat-Barros R. (2018). Effects of prefrontal transcranial direct current stimulation and motivation to quit in tobacco smokers: a randomized, sham controlled, double-blind trial. *Front. Pharmacol.* 9:14. 10.3389/fphar.2018.00014 29434547 PMC5791546

[B145] Volpato C. Piccione F. Cavinato M. Duzzi D. Schiff S. Foscolo L. (2013). Modulation of affective symptoms and resting state activity by brain stimulation in a treatment-resistant case of obsessive-compulsive disorder. *Neurocase* 19 360–370. 10.1080/13554794.2012.667131 22554168

[B146] Vossel S. Geng J. J. Fink G. R. (2014). Dorsal and ventral attention systems: distinct neural circuits but collaborative roles. *Neuroscientist* 20 150–159. 10.1177/1073858413494269 23835449 PMC4107817

[B147] Weickert T. W. Salimuddin H. Lenroot R. K. Bruggemann J. Loo C. Vercammen A. (2019). Preliminary findings of four-week, task-based anodal prefrontal cortex transcranial direct current stimulation transferring to other cognitive improvements in schizophrenia. *Psychiatry Res.* 280:112487. 10.1016/j.psychres.2019.112487 31376788

[B148] Welch E. S. Weigand A. Hooker J. E. Philip N. S. Tyrka A. R. Press D. Z. (2019). Feasibility of computerized cognitive-behavioral therapy combined with bifrontal transcranial direct current stimulation for treatment of major depression. *Neuromodulation* 22 898–903. 10.1111/ner.12807 30153360

[B149] Wietschorke K. Lippold J. Jacob C. Polak T. Herrmann M. J. (2016). Transcranial direct current stimulation of the prefrontal cortex reduces cue-reactivity in alcohol-dependent patients. *J. Neural. Transm (Vienna).* 123 1173–1178. 10.1007/s00702-016-1541-6 27038632

[B150] Woods A. J. Antal A. Bikson M. Boggio P. S. Brunoni A. R. Celnik P. (2016). A technical guide to tDCS, and related non-invasive brain stimulation tools. *Clin. Neurophysiol.* 127 1031–1048. 10.1016/j.clinph.2015.11.012 26652115 PMC4747791

[B151] Wrigley P. J. Gustin S. M. McIndoe L. N. Chakiath R. J. Henderson L. A. Siddall P. J. (2013). Longstanding neuropathic pain after spinal cord injury is refractory to transcranial direct current stimulation: a randomized controlled trial. *Pain* 154 2178–2184. 10.1016/j.pain.2013.06.045 23831866

[B152] Wu L. L. Potenza M. N. Zhou N. Kober H. Shi X. H. Yip S. W. (2020). A role for the right dorsolateral prefrontal cortex in enhancing regulation of both craving and negative emotions in internet gaming disorder: a randomized trial. *Eur. Neuropsychopharmacol.* 36 29–37. 10.1016/j.euroneuro.2020.04.003 32446706 PMC8292795

[B153] Xu J. Fregni F. Brody A. L. Rahman A. S. (2013). Transcranial direct current stimulation reduces negative affect but not cigarette craving in overnight abstinent smokers. *Front. Psychiatry* 4:112. 10.3389/fpsyt.2013.00112 24065930 PMC3778370

[B154] Xu X. Deng Z. Y. Huang Q. Zhang W. X. Qi C. Z. Huang J. A. (2017). Prefrontal cortex-mediated executive function as assessed by stroop task performance associates with weight loss among overweight and obese adolescents and young adults. *Behav. Brain Res.* 321 240–248. 10.1016/j.bbr.2016.12.040 28043899

[B155] Yang X. Gao M. Shi J. Ye H. Chen S. (2017). Modulating the activity of the DLPFC and OFC has distinct effects on risk and ambiguity decision-making: a tDCS study. *Front. Psychol.* 8:1417. 10.3389/fpsyg.2017.01417 28878714 PMC5572270

[B156] Yoon Y. B. Kim M. Lee J. Cho K. I. K. Kwak S. Lee T. Y. (2019). Effect of tDCS on aberrant functional network connectivity in refractory hallucinatory schizophrenia: a pilot study. *Psychiatry Investig.* 16 244–248. 10.30773/pi.2018.11.18 30836741 PMC6444100

